# Challenges to Safe Nanomedicine Treatment

**DOI:** 10.3390/nano13071171

**Published:** 2023-03-25

**Authors:** Chunhua Yang, Didier Merlin

**Affiliations:** 1 Digestive Disease Research Group, Institute for Biomedical Sciences, Georgia State University, Atlanta, GA 30303, USA; dmerlin@gsu.edu; 2 Atlanta Veterans Affairs Medical Center, Decatur, GA 30033, USA

Nanotechnology has the potential to revolutionize the field of drug treatment by enabling the targeted delivery and controlled release of drugs at a cellular level. For example, by using antibodies or peptides that can bind to cancer-associated transmembrane receptors, surface-modified nanoparticles (NPs) can specifically target cancer cells, increasing the efficacy of cancer treatments while reducing side effects [1]. In addition, NPs can be designed to release drugs in a controlled manner over time, which can reduce the frequency of dosing [2]. Smart nanomedicines can also cross biological barriers (such as the blood–brain barrier) and reach specific sites in the body to enhance the loaded drug’s potency [3]. 

Despite their tremendous potential, nanomedicine-based treatments are facing several safety challenges. Because nanomedicines are significantly smaller and have much higher specific surface areas than traditionally formulated drugs, they interact with cells and tissues in ways that are not yet fully understood, making it difficult to predict and prevent potential toxicities. Studies have highlighted the multilateral nature of nanomedicine-derived toxicity, showing that NPs induce oxidative stress, immune responses, and inflammation, and also cause genotoxicity and irreversible alterations to the intracellular organelles [4]. In addition, orally administered NPs may be detrimental to the gut barrier or alter gut microbiome homeostasis [5,6]. 

In the complex context of NP-induced oxidative stress, it is necessary to monitor changes in the concentration of free radicals, such as reactive oxygen and nitrogen species (RONS), during treatment [7]. An imbalance between the production and elimination of RONS, especially the excessive regional accumulation of RONS, causes damage to intracellular lipids, proteins, and DNA [8]. Such damage can trigger unpredicted chain reactions that cause immunogenicity and acute inflammation. For example, some metal nanoparticles (MNPs) catalyze the production of oxygen-derived metabolites and reactive hydroxyl radicals that induce lipid peroxidation [9]. Intracellular lipid peroxidation can further cause the malfunction of multiple organelles, including the endoplasmic reticulum (ER), mitochondria, and plasma membrane [10]. 

Nanomedicine-induced oxidative stress also involves actions that obstruct the RONS-eliminating functions of cells. Normally, cells produce reduced glutathione (GSH), α-lipoic acid, and superoxide dismutase (SOD) as protective mechanisms for neutralizing excess RONS. Metal oxide nanoparticles (MONPs), such as copper-derived MONPs, decrease the cellular level of GSH and disturb mitochondrial transmembrane potential, directly affecting the antioxidant-protective function of macrophages [11]. Orally delivered MONPs, such as TiO_2_-NPs, were found to affect the ratio of GSH to oxidized glutathione (GSSG) in hepatic cells, elevating the ROS concentration sufficiently enough to cause lipid peroxidation and to induce a corresponding increase in micronucleus frequency—a sign of potential genotoxicity [12]. Other studies have shown that ZnO-NPs (50 mg/kg) significantly disturb SOD generation and inducing severe intestinal injuries in rats [13].

Apart from impacting intracellular redox homeostasis, nanomedicine treatment is associated with intracellular organelle toxicity. Once engulfed by cells, NPs engage the endosome–lysosome degradation pathway. Generally, the surface charge of NPs determines their degradative fate. Negatively charged NPs are trapped in endosomes and delivered to lysosomes, where some NPs induce lysosome membrane permeabilization (LMP). LMP potentially causes cytosolic acidification and initiates the disintegration of cellular components, and induces apoptosis [14]. In contrast, positively charged NPs can generally escape from the endosome–lysosome pathway: a property that has been harnessed for the application of these NPs for intracellular drug delivery. However, even in this case, caution is warranted due to the off-target effects of these escaped NPs, which also induce damage to organelles, such as the ER and mitochondria. For example, the so-called “ER stress” pathway can be activated by the NP-induced accumulation of unfolded proteins [15], which, in turn, can trigger the accumulation of autophagosomes and activate the caspase-independent cell death pathway, leading to autophagic cell death [16].

The genotoxicity of nanomedicines reflects NP-induced damage to genetic materials. These include DNA strand breaks, point mutations, or even chromosomal fragmentation and rearrangement, which can lead to cancer and other negative health effects. The direct genotoxicity of nanomedicines is caused mostly by interactions between NPs and chromosomes during the interphase, with ultrafine NPs binding to DNA molecules and preventing DNA replication or transcription. Graphene oxide (GO) NPs were found to directly induce DNA damage in the intestinal cells of Drosophila, causing various behavioral and developmental defects in offspring [17]. Indirect genotoxicity is typically related to NP-induced ROS formation or the release of toxic ions. These toxins interfere with the proteins responsible for normal genetic functions, such as DNA replication, transcription, or repair. ROS can cause the oxidization of purines and pyrimidines, leading to mispairing and consequent destructive mutations. Decreasing the size of nanomedicines, which increases their surface area to volume ratio, can induce higher ROS production, thereby further increasing the possibility of genotoxicity. 

As we continue to discover the mechanisms through which nanomedicines interact with biological systems, we can also learn about their potential risk of immunotoxicity. An immune response to nanomedicine occurs when the body’s immune system recognizes NPs as foreign invaders and attacks them, potentially causing regional inflammation. Not all nanomaterials are immunotoxic, and the potential for immunotoxicity can vary depending on the specific nanomaterial and the dose and duration of exposure. It is important to note that, even using biocompatible polymer-based NPs, there is a risk of triggering immunotoxicity. For example, although the free poly(ethylene glycol) (PEG) polymer exhibits little to no immunogenicity, it tends to become immunogenic once attached to certain proteins, evoking PEG-specific antibody (Ab) responses, similar to a hapten [18]. Clinical reports have shown that anti-PEG Abs can cause the failure of PEGylated nanomedicine treatments and even result in lethal adverse effects [18]. Other biodegradable polymeric nanomaterials can display toxicity in association with their degradation [19]. As their physicochemical properties and concentration change during polymer degradation, NPs may evoke evolving immunogenicity. For some nanomaterials, there is also a chance of NP-induced hypersensitivity reactions—allergic-like responses that lead to symptoms such as hives, rash, itching, and difficulty breathing [20]. 

At this point, our understanding of the long-term effects of exposure to nanomaterials remains limited. Long-term toxicity may result in serious adverse effects in late-stage trials. The constant oral administration of nanomedicines could potentially affect the gut barrier, even if the corresponding NPs are constructed from FDA-approved, safe materials. For example, chitosan NPs are generally safe, but the long-term use of chitosan nanomaterials can cause the excessive absorption of cations, inducing calcium (Ca^2+^) and magnesium (Mg^2+^) defects and affecting the formation of tight junctions [21]. Studies have also shown that the long-term consumption of MNPs or MONPs in popular consumer products can shift a healthy gut microbiome towards an abnormal microbiome, suggesting that gut microbiota are involved in the pathway that connects NPs to long-term toxicity [6,22].

It is worth noting that the pharmacokinetics and pharmacodynamics of nanomaterials are easily overlooked in studies of the distribution and localization of nanomedicines, which focus primarily on the delivered drug, not the nanomaterials. Due to limitations in detection methods, it is difficult to monitor the metabolism and disposition of polymeric nanomaterials in the body. In fact, some large-sized (~200 nm) polymeric nanomaterials tend to accumulate in certain tissues or organs, such as the liver and spleen, ultimately interfering with drug metabolism and transport and affecting their efficacy and safety [23]. In this context, it is important to closely monitor patients who have received these treatments and conduct post-market surveillance to identify any potential adverse effects. Importantly, safety monitoring is a continuous process, and as new technologies and information emerge, surveillance protocols should be updated accordingly.

Developing safe and effective nanomedicine treatments for disease requires a multidisciplinary approach that involves collaboration among scientists, engineers, clinicians, and regulatory agencies. Therefore, at the current stage of development, nanomedicines can be costly to produce, making it difficult for most patients to access them. Although regulations governing the application of nanomedicine are still developing because the technology is relatively new, some key regulatory bodies have been closely overseeing the development and use of nanomedicines. The US Food and Drug Administration (FDA), the European Medicines Agency (EMA), the World Health Organization (WHO), and the International Council for Harmonization of Technical Requirements for Pharmaceuticals for Human Use (ICH) are actively involved in establishing and renewing regulations related to the development of nanomedicines. We believe that, with the development of preclinical and clinical safety regulations and the advancement of nanotechnology, nanomedicines will become safer and more accessible for most patients.
